# Identification and Validation of a Potential Marker of Tissue Quality Using Gene Expression Analysis of Human Colorectal Tissue

**DOI:** 10.1371/journal.pone.0133987

**Published:** 2015-07-29

**Authors:** Nicole Lange, Florian T. Unger, Monika Schöppler, Katja Pursche, Hartmut Juhl, Kerstin A. David

**Affiliations:** Indivumed GmbH, Hamburg, Germany; Sun Yat-sen University Cancer Center, CHINA

## Abstract

Correlative studies have identified numerous biomarkers that are individualizing therapy across many medical specialties, including oncology. Accurate interpretation of these studies requires the collection of tissue samples of sufficient quality. Tissue quality can be measured by changes in levels of gene expression and can be influenced by many factors including pre-analytical conditions, ischemic effects and the surgical collection procedure itself. However, as yet there are no reliable biomarkers of tissue quality at researchers’ disposal. The aim of the current study was to identify genes with expression patterns that fluctuated reproducibly in response to typical post-surgical stress (ischemia) in order to identify a specific marker of tissue quality. All tissue samples were obtained from patients with primary colorectal carcinoma (CRC) (N = 40) either via colonoscopy prior to surgery, or by surgical resection. Surgically resected tissue samples were divided into three groups and subjected to cold ischemia for 10, 20 or 45 minutes. Normal colorectal tissue and CRC tissue was analyzed using microarray and quantitative real-time PCR (qPCR). Comparing changes in gene expression between pre- and post-surgical tissue using microarray analysis identified a list of potential tissue quality biomarkers and this list was validated using qPCR. Results revealed that post-operative ischemia significantly alters gene expression in normal and CRC tissue samples. Both microarray analysis and qPCR revealed regulator of G-protein signaling 1 (*RGS1*) as a potential marker of CRC tissue quality and eukaryotic translation elongation factor 1 alpha 1 (*EEF1A1*) as a potential reference gene of post-operative tissue quality. Larger studies with additional time points and endpoints will be needed to confirm these results.

## Introduction

In the field of cancer research the collection of tissue samples and, in particular, the quality of tissue samples is integral to the identification of prognostic and predictive biomarkers **[1]**. Many factors can influence tissue sample quality, including pre-analytical conditions (collection time and ischemic effects), exogenous factors, and the type of surgical collection procedure itself **[2,3,4]**. The quality of a tissue sample can in turn influence gene expression profiles, and the molecular composition of cells, tissues and fluids. Understanding the impact of tissue sample quality on gene expression is crucial in order to avoid data misinterpretation. Identifying specific, validated biomarkers that indicate tissue of high quality would benefit future research.

In this study, we conducted a systematic analysis of the impact of post-operative ischemia on normal and tumor colorectal tissue samples from patients with colorectal cancer (CRC), in order to identify and evaluate a potential tissue-quality biomarker. Tissue collection was performed in a highly standardized manner and included a colonoscopy sample removed immediately before surgery, as well as tissue samples that underwent cold ischemia 10, 20 and 45 minutes post-surgical resection. In a previous study by David and colleagues **[5]**, gene-expression analysis of these tissue samples was performed using microarray technology followed by hierarchical clustering and analysis of variance (ANOVA) examination. Results yielded a list of target genes that were similarly regulated in patient samples following resection and following cold ischemia time.

Using the target genes identified in the study by David *et al* **[5]**, the aim of the current study was to use quantitative real-time PCR (qPCR) to identify genes with an expression pattern that fluctuated reproducibly in response to cold ischemia with a view to identify a specific marker of tissue quality. Results revealed that post-operative ischemia significantly affects gene expression in normal and tumor colorectal tissue samples. Both microarray analysis and qPCR revealed regulator of G-protein signaling 1 (*RGS1)* as a potential marker of colorectal tissue quality and eukaryotic translation elongation factor 1 alpha 1 (*EEF1A1)* as a potential reference gene of post-operative tissue quality.

## Materials and Methods

### Patient enrollment

Patients with primary CRC and tumors larger than 3 cm in diameter were enrolled. Patients who had received chemotherapy or radiation therapy less than 3 weeks before surgery were excluded. The study was conducted at two different sites in Hamburg, Germany, and reviewed and approved by the competent ethics review committee of the Medical Association of Hamburg, Germany under reference number PV3342. Prior to this study, all patients gave their written informed consent.

### Tissue collection before and during surgery

After induction of anesthesia, patients underwent colonoscopy, upon which three biopsies were taken from the tumor (T) and three biopsies were taken from the adjacent normal tissue (N). Throughout this manuscript, this time point will be designated as 0. After resection of the tumor and adjacent normal tissue, 12 tissue samples were collected from the tumor tissue and 12 tissue samples were collected from the adjacent normal tissue at room temperature and standard air ratio (21% oxygen). These 24 tissue samples from tumor and normal tissue were divided into three groups and exposed to a cold ischemia time of 10 minutes (10), 20 minutes (20) or 45 minutes (45). Cold ischemia time reflected the time interval between removing the tissue and preserving tissue samples **[2,3]**. Every tissue sample was approximately 5 x 5 x 5 mm and 120 mg. For each of the time points and each tissue type (normal or tumor), half of the samples were immediately stored in the vapor phase of liquid nitrogen, and half were immersion-fixed in 4% formaldehyde. In this study, only frozen tissue samples were used.

### Microarray analysis

Oligonucleotide microarray analyses (GeneChip Human Genome U133 Plus 2.0; Affymetrix Inc., Santa Clara, CA, USA) were performed as described previously **[5]**. Complete data sets including all time points from 40 patients were analyzed. Data revealed a similar pattern of gene expression changes for most of the patients **[5]**.

### Preparation of RNA and RNA quality control

For qPCR analysis, RNA was extracted from normal (tumor content 0%) and tumor (tumor content ≥50%) tissue samples. Tissue sample RNA was isolated by performing a phenol-chloroform extraction (QIAzol), followed by a DNase digestion and high-quality purification using the RNeasy MinElute Cleanup Kit. Reagents and kits were purchased from Qiagen (Hilden, Germany) and purification procedures were performed according to the manufacturer’s protocols. RNA concentration was measured using the NanoDrop 2000 spectrophotometer (Thermo Fisher Scientific, Dreieich, Germany). RNA quality was determined by measuring the RNA integrity number (RIN) using the RNA 6000 Nano Kit and a bioanalyzer (Agilent Technologies, Berlin, Germany), according to the supplier’s instructions. A minimum RIN of ≥6 was mandatory for extracted RNA to pass the quality control.

### cDNA synthesis

cDNA synthesis from extracted RNA, as well as synthesis of non-reverse transcriptase controls, were performed using the QuantiTect Reverse Transcription Kit (Qiagen, Hilden, Germany) according to the supplier’s instructions. The QuantiTect Reverse Transcription Kit included a blend of oligo(dT) and random primers and an additional step for the elimination of contaminating genomic DNA. Each sample contained 1 μg of template RNA in a reaction volume of 20 μl.

### Detection of mRNA expression by qPCR

qPCR analysis was performed using the 2x SsoAdvanced Universal SYBR Green Supermix as well as the PrimePCR SYBR Green Assays (both Bio-Rad, Munich, Germany) containing unlabeled oligonucleotide primer pairs and were used according to the supplier’s instructions. Amplification was carried out in a reaction volume of 20 μl using cDNA generated from 10 ng of RNA on the Bio-Rad C1000 Touch Thermal Cycler (CFX Manager Software Version 3.0; Munich, Germany). The thermal cycling protocol included a single polymerase activation step at 95°C for 2 minutes followed by 40 amplification cycles as well as melt-curve analysis. Each amplification cycle comprised a denaturation step at 95°C for 10 seconds and a primer annealing/elongation step at 60°C for 30 seconds. Melt-curve analysis was performed including a 30-second hold at 65°C followed by a gradual increase to 95°C with a temperature increment of 0.5°C every 5 sec. Primer efficiency was determined by performing standard curve analysis. Furthermore, intra-assay variation (repeatability) and inter-assay variation (reproducibility) were assayed. According to the minimum information for publication of qPCR experiments (MIQE) guidelines **[6]**, all information including RNA, cDNA and qPCR analyses (e.g. conditions, reagents, quality rules) were summarized within the MIQE-checklist (**S1–S3 Tables**).

### Data analyses and statistical analyses

Gene expression data obtained with the C1000 Touch Thermal Cycler and CFX Manager Software Version 3.0 (Bio-Rad, Munich, Germany) were exported to Microsoft Office Excel 2007 and further analyzed. For relative quantification of mRNA expression the ΔΔCq method was applied. Results were statistically analyzed using GraphPad Prism 5.0 (GraphPad Software, Inc.; San Diego, CA, USA). Descriptive column statistics of each data set were performed, including the Shapiro–Wilk normality test. Non-Gaussian distribution of data sets (p≤0.05) were tested for significant differences using the Kruskal–Wallis test in combination with the Dunn’s multiple comparison post test and Gaussian distribution of data sets (p>0.05) was tested using one-way ANOVA in combination with the Dunnett’s multiple comparison post test. The significance level was ≤0.05. All numerical data and statistical analyses were summarized in **S1 Appendix**.

## Results

### Patient disposition and clinical data

Based on the microarray data of the previous published study **[5]** normal colorectal tissue and CRC tissue samples were analyzed to identify gene expression changes in response to cold ischemia. In total, 50 patients with CRC who were scheduled for tumor resection surgery gave informed consent to be enrolled in the study; complete and evaluable microarray results were available from 40 patients (n = 21 female, n = 19 male; 44 to 89 years [**S4 Table**]). Medical examination revealed malignant neoplasms of the rectum (n = 22), of the sigmoid colon (n = 16), of the descending colon (n = 1) and carcinoma *in situ* of the rectum (n = 1). Cancer staging revealed: n = 8/stage I; n = 10/stage II; n = 8/stage III and n = 11/stage IV (two tumors were not applicable and one was stage 0). Surgical resection was performed in two hospitals (16 patients/Alten Eichen-Hospital Hamburg; 24 patients/Israelite Hospital Hamburg). These clinical data revealed a sufficiently large and heterogeneous group of patients for further analysis.

### Microarray data evaluation

**Fig 1:**
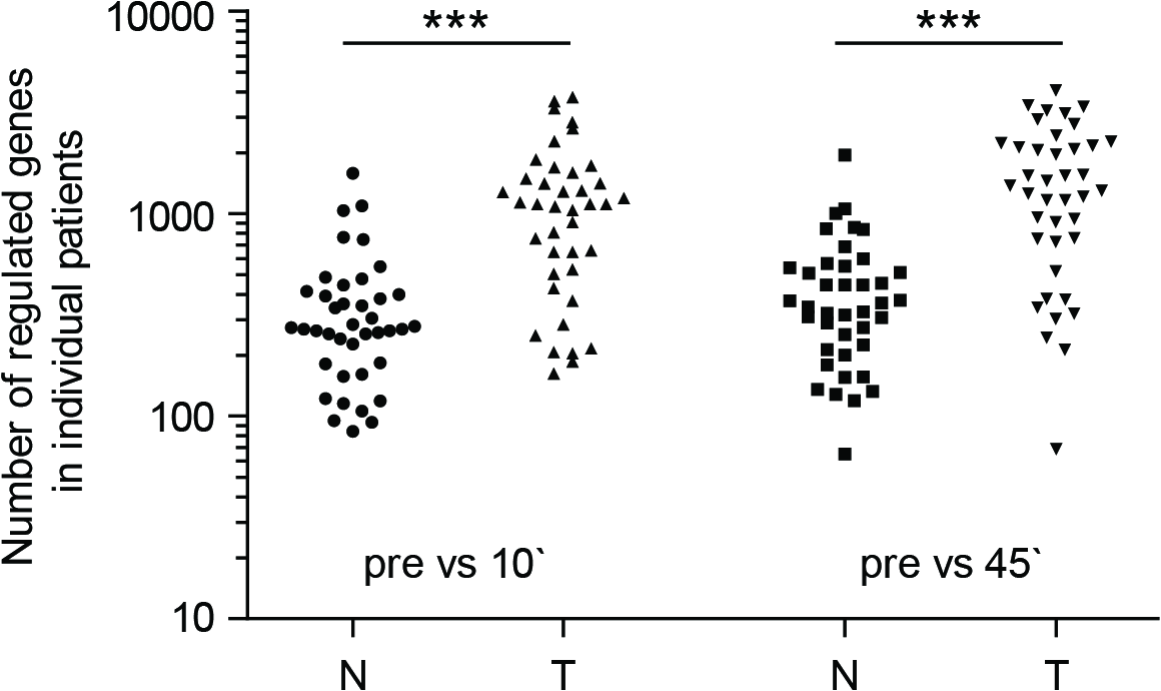
Number of regulated genes in colon tissue summarized from microarray data. Number of genes in 40 individual patients that change expression > 2-fold, comparing different time points of collection: N = normal tissue; T = tumor tissue; pre = before surgery; 10` = 10 minutes after resection; 45` = 45 minutes after resection. Kruskal-Wallis test and Dunn’s multiple comparison test were used for statistical analysis. *** p ≤ 0.001.

Data were analyzed from colonoscopy samples (N0 or T0) and 10-, 20- and 45-minute cold ischemia samples from normal as well as tumor tissue. The data sets of 40 patients passed quality rules and were analyzed. The evaluation of microarray data included the comparison of the pre-surgical gene expression profiles to the corresponding gene expression profiles 10 and 45 minutes post-resection. **Fig 1**show genes from 40 patients exhibiting a ≥2-fold change in gene expression. The number of up- or down-regulated genes varied between patients in each group, however, gene expression changes in response to resection were significantly higher in tumor tissue compared with normal tissue. While ≤3792 genes were regulated in tumor tissue only ≤1584 genes were regulated in normal tissue samples 10 minutes post-resection (p<0.001). Similar results were obtained when comparing samples collected before surgery with samples collected 45 minutes post-surgery (tumor tissue: ≤4116 genes; normal tissue: ≤1956 genes p<0.001).

As described previously **[5],** hierarchical clustering and comparison of log-fold changes between normal and tumor ischemia time points were performed. Of these, five potential tissue biomarker candidates were chosen (**Table 1**). Gene expression differences of log-fold change ≥2 were identified for *CYR61*, *RGS1*, *DUSP1*, *DUOX2* and *SLC6A14* in normal tissue by comparing pre-surgical to 45 minutes post-surgical time points. Corresponding tumor tissue revealed similar, but generally lower, log-fold changes in gene expression. To identify potential reference genes, the coefficients of variation (CV) across ischemic time points were determined. Probe sets with the lowest CVs in normal tissue are listed in **Table 2**. *EEF1A1* exhibited a small CV and the best correlation of ranks between normal and tumor tissue.

**Table 1:** Microarray data of differentially expressed genes in normal and colorectal tumor tissue.

			Normal colorectal tissue				Colorectal tumor tissue			
Probe ID	Gene Symbol	Protein	Comparison pre vs 10’		Comparison pre vs 45’		Comparison pre vs 10’		Comparison pre vs 45’	
** **	** **	** **	p-value	**Log-fold Change**	p-value	**Log-fold Change**	p-value	**Log-fold Change**	p-value	**Log-fold Change**
201289_at	*CYR61*	Cysteine-rich angiogenic inducer 61	1.57E-18	**2.49**	2.84E-22	**2.55**	1.33E-06	**1.41**	3.96E-08	**1.59**
202988_s_at	*RGS1*	Regulator of G-protein signaling 1	4.21E-21	**2.34**	5.05E-25	**2.39**	1.81E-09	**1.57**	7.62E-12	**1.91**
216834_at	*RGS1*	Regulator of G-protein signaling 1	1.65E-22	**2.33**	8.96E-25	**2.36**	3.06E-09	**1.42**	5.20E-12	**1.76**
201041_s_at	*DUSP1*	Dual specificity phosphatase 1	1.21E-17	**1.85**	3.00E-23	**2.15**	–	**–**	1.15E-05	**1.14**
219727_at	*DUOX2*	Dual oxidase 2	3.36E-08	**-1.88**	2.24E-10	**-2.14**	4.97E-04	**-1.51**	1.33E-05	**-1.82**
219795_at	*SLC6A14*	Solute carrier family 6 (amino acid transporter), member 14	1.01E-05	**-1.87**	3.60E-07	**-2.16**	1.09E-02	**-1.2**	3.16E-04	**-1.75**

Gene expression was compared before surgery (pre) and 10 minutes after resection (10’), and Pre and 45 minutes after resection (45’). Table adapted from David K et al. Oncotarget 2014; 5. **[5]**.

**Table 2:** Microarray data of genes with the lowest CV across ischemia time points in normal colorectal tissue.

			Normal tissue		Tumor tissue
Probe ID	Gene	Protein	CV	Rank	Rank
1558623_at	*LOC729121*	Hypothetical LOC729121	0.000065177	1	22,547
227472_at	*DDA1*	DET1 and DDB1 associated 1	0.000076330	2	5,095
213477_x_at	*EEF1A1*	Eukaryotic translation elongation factor 1 alpha 1	0.000120035	3	70
206559_x_at	*EEF1A1*	Eukaryotic translation elongation factor 1 alpha 1	0.00021194	10	464
203172_at	*FXR2*	Fragile X mental retardation, autosomal homolog 2	0.00012495	4	5,833
202652_at	*APBB1*	Amyloid beta (A4) precursor protein-binding, family B, member 1	0.000140598	5	22,323
209394_at	*ASMTL*	Acetylserotonin O-methyltransferase-like	0.000153558	6	28,176
234891_at	*DKFZP547L112*	Hypothetical protein DKFZp547L112	0.000198356	7	17,884
212986_s_at	*TLK2*	Tousled-like kinase 2	0.000208644	8	2,237
223148_at	*PIGS*	Phosphatidylinositol glycan anchor biosynthesis, class S	0.000210952	9	24,752
212581_x_at	*GAPDH*	Glyceraldehyde-3-phosphate dehydrogenase	0.001735025	3208	332
211296_x_at	*UBC*	Ubiquitin C	0.003508935	16,259	2,04

The 10 probe sets (nine genes) with the lowest CV including rank across all four time points (pre-surgery, 10, 20 and 45 minutes after resection) in normal colorectal tissue as well as the corresponding ranks in tumor colorectal tissue are shown. Additionally, CVs and ranks of *GAPDH* and *UBC* are included within this table. Table adapted from David K et al. Oncotarget 2014; 5. **[5]**.

### RNA integrity and qPCR analysis

**Fig 2:**
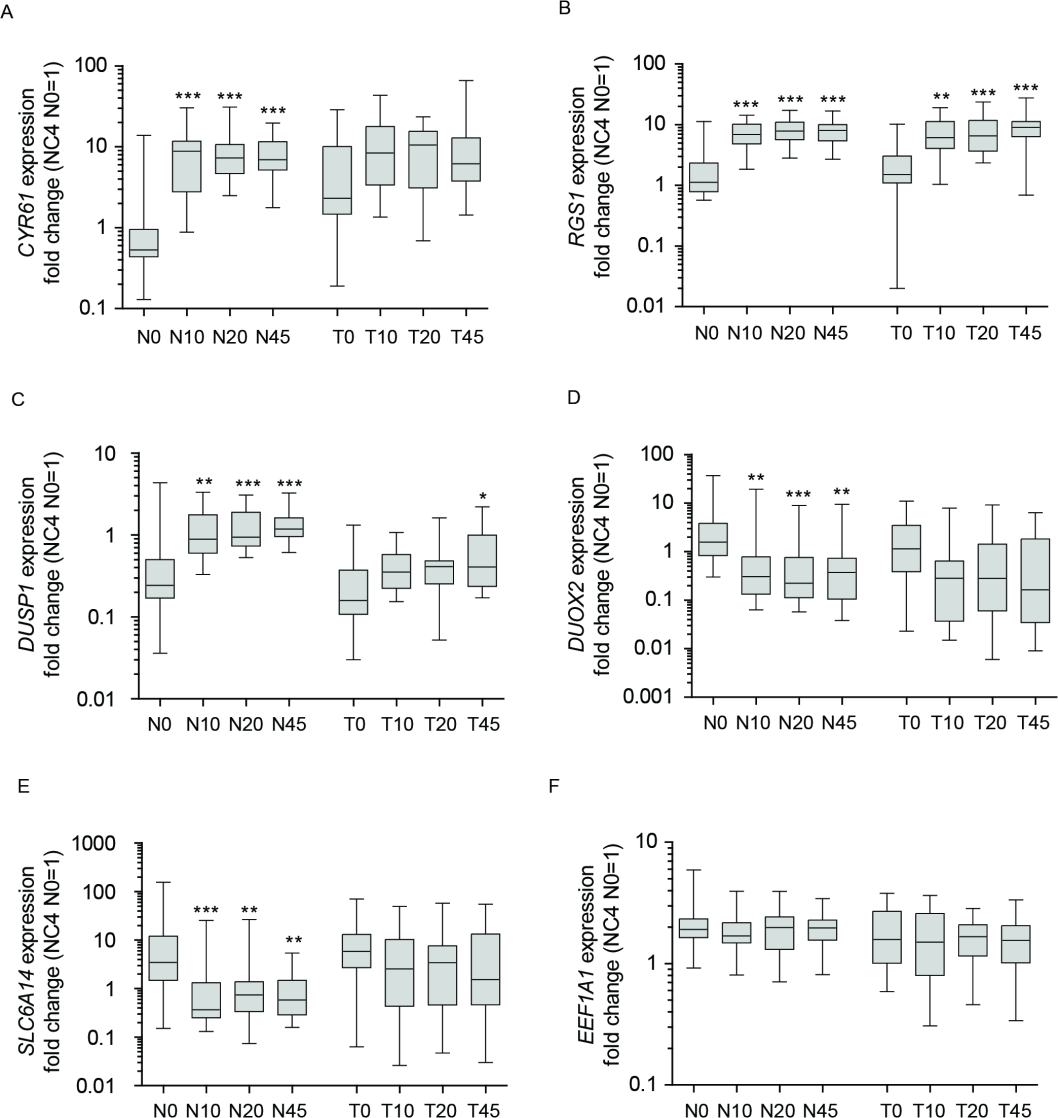
qPCR gene expression analysis of six target genes in response to ischemia. Time-dependent gene expression of *CYR61* **(A)**, *RGS1* **(B)**, *DUSP1* **(C)**, *DUOX2* **(D)**, *SLC6A14* **(E)** and *EEF1A1* **(F)** in normal (N) compared with tumor (T) tissue from 20 patients shown in Box-Whisker Plots. Boxes represent first and third quartile, whiskers represent minima and maxima, solid lines within boxes indicate medians. Results are displayed as fold changes normalized to sample NC4/N0. Kruskal-Wallis test and Dunn’s multiple comparison test were used for statistical analysis. Significant changes in gene expression between N0 and N10/N20 or N45 as well as between T0 and T10/T20 or T45 are indicated N = normal tissue; T = tumor tissue; 0 = before surgery; 10, 20, 45 = 10, 20, 45 minutes after resection; * p ≤ 0.05; ** p ≤ 0.01; *** p ≤ 0.001.

To validate the microarray results a qPCR assay was developed and the mRNA expression of six chosen genes (*CYR61*, *RGS1*, *DUOX2*, *SLC6A14*, *EEF1A1* and *DUSP1)* that fluctuated reproducibly in normal and tumor colorectal tissue groups in response to ischemia were analyzed. As it is well known that degraded RNA negatively influences the outcome of qPCR analyses, RNA quality was determined. Individual as well as mean RIN values from all patient samples are shown in **S1 Fig**. Mean RIN values were high (RIN ≥7.9) and 88.75% of individual samples (142/160) showed RIN values ≥7.5 threshold. Furthermore, 11.25% samples (18/160) showed RIN values of between 5 and 7.5. RNA was therefore of sufficient quality for reliable qPCR quantification **[7]**. The stably expressed reference genes, *GAPDH* and *UBC* **[8]**, were used for normalization of the qPCR results. **Fig 2**shows the changes in expression of the six target genes. Consistent with the microarray results, qPCR analysis revealed a statistically significant up-regulation of *CYR61*, *DUSP1* and *RGS1* as well as a statistically significant down-regulation of *SLC6A14* and *DUOX2* in normal ischemic tissue samples compared with the pre-surgical sample (**Fig 2**). When gene expression was compared between ischemic time points (N10/N20; N10/N45; N20/N45) no differences were observed. Furthermore, expression of *EEF1A1* was unchanged when comparing expression in normal and tumor tissue samples after resection (**Fig 2F**). In contrast, the results obtained from tumor tissue samples were more heterogeneous. Although expression of *CYR61*, *DUSP1* and *RGS1* was detected, only *RGS1* showed significant up-regulation in tumor tissue across all time points of ischemia (**Fig 2B**). Furthermore, statistical analysis revealed no significant decrease in the expression of *SLC6A14* and *DUOX2* in response to ischemia.

Additionally, gene expression changes between normal and tumor colonoscopy samples (N0/T0) were analyzed for all genes. Within this study, no statistically significant gene expression changes were found, although an increased but not significant expression of *CYR61* was observed in tumor tissue (T0) compared with normal tissue (N0) (**Fig 2A**).

### Focus on *RGS1* and *EEF1A1* qPCR data

**Fig 3:**
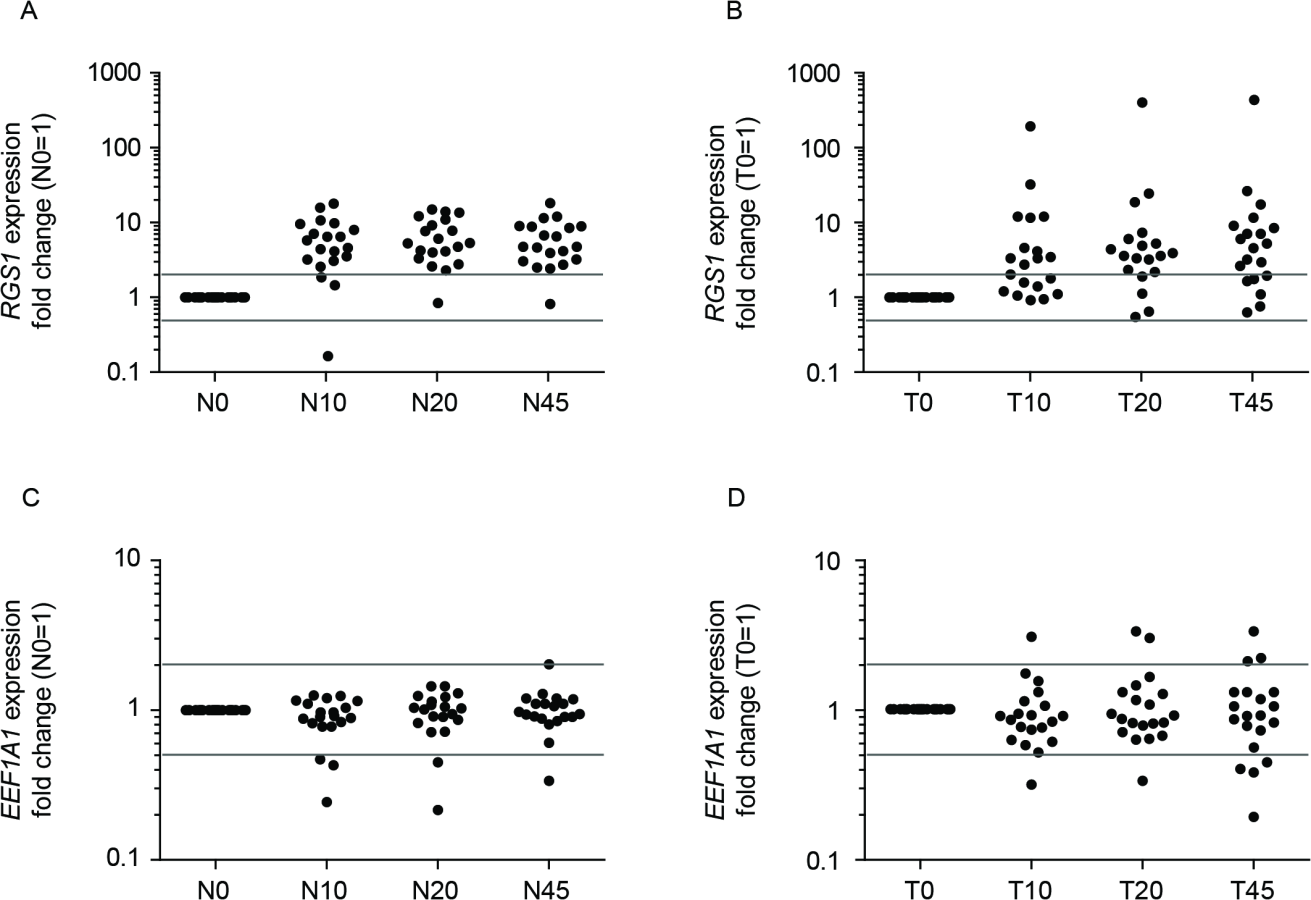
Regulation of *RGS1* and *EEF1A1* expression levels in colorectal ischemic samples. Comparison of *RGS1* expression changes in normal (**A**) and tumor **(B)** ischemic tissue samples and *EEF1A1* expression changes in normal **(C)** and tumor **(D)** ischemic tissue samples of 20 individual patients. Patients pre-surgery samples (0) were set to 1 and individual fold changes of ischemic samples were calculated and displayed as dots. Grey lines indicate a 2-fold change in gene expression compared with individual pre-surgical samples. N = normal tissue; T = tumor tissue; 0 = before surgery; 10, 20, 45 = 10, 20, 45 minutes after resection.

To investigate the expression of *RGS1* and *EEF1A1* in more detail, fold-changes in gene expression in ischemic tissue samples were assessed, and 2-fold changes indicated (**Fig 3**). *RGS1* expression increased more than 2-fold in nearly all normal tissue samples (N10: 17/20 patients; N20: 19/20; N45: 19/20) as well as in the majority of tumor tissue samples (T10: 12/20; T20: 15/19; T45: 14/20). In normal tissue samples, *RGS1* expression was at its highest within 10 minutes post-resection and expression appeared constant across all ischemia time points (**Fig 3A**). In tumor tissue samples, *RGS1* expression level increased over time and was stable 20 minutes post-resection (**Fig 3B**). Time-dependent regulation of *EEF1A1* in normal and tumor tissue resulted in stable expression (≤2-fold change) of *EEF1A1* in nearly all samples (**Fig 3C and 3D**; N10: 17/20; N20: 18/20; N45: 19/20; T10: 18/20; T20: 17/20; T45: 13/20). However, the distribution of individual values within each group revealed a more focused clustering in response to resection in normal compared with tumor tissue samples.

### *EEF1A1* as a reference gene

To investigate the potential of *EEF1A1* to serve as a reference gene, the CV of the reference genes GAPDH and UBC was compared with the proposed reference gene, *EEF1A1* (**Table 3**). *EEF1A1* was associated with the lowest CV (*EEF1A1*: 3.63) in all normal tissue samples. In tumor tissue samples, the CV of *EEF1A1* (6.12) was the second highest compared with *UBC* and *GAPDH*.

**Table 3:** CVs of *GAPDH*, *UBC* and *EEF1A1* across ischemia time points and patients in normal and tumor colorectal tissue.

	Normal tissue			Tumor tissue		
	Mean	SD	CV	Mean	SD	CV
	[Cq value]		[%]	[Cq value]		[%]
***GAPDH***	20.81	1.04	4.99	19.85	1.42	7.14
***UBC***	22.71	1.31	5.77	22.41	1.28	5.71
***EEF1A1***	29.01	1.05	3.63	28.73	1.76	6.12

Mean value of *GAPDH*, *UBC* and *EEF1A1* Cq values as well as standard deviation (SD) across all patients and time points were calculated. Furthermore, the CV across all four time points (pre-surgery, 10, 20 and 45 minutes after resection) in normal and tumor colorectal tissue was calculated.

**Fig 4:**
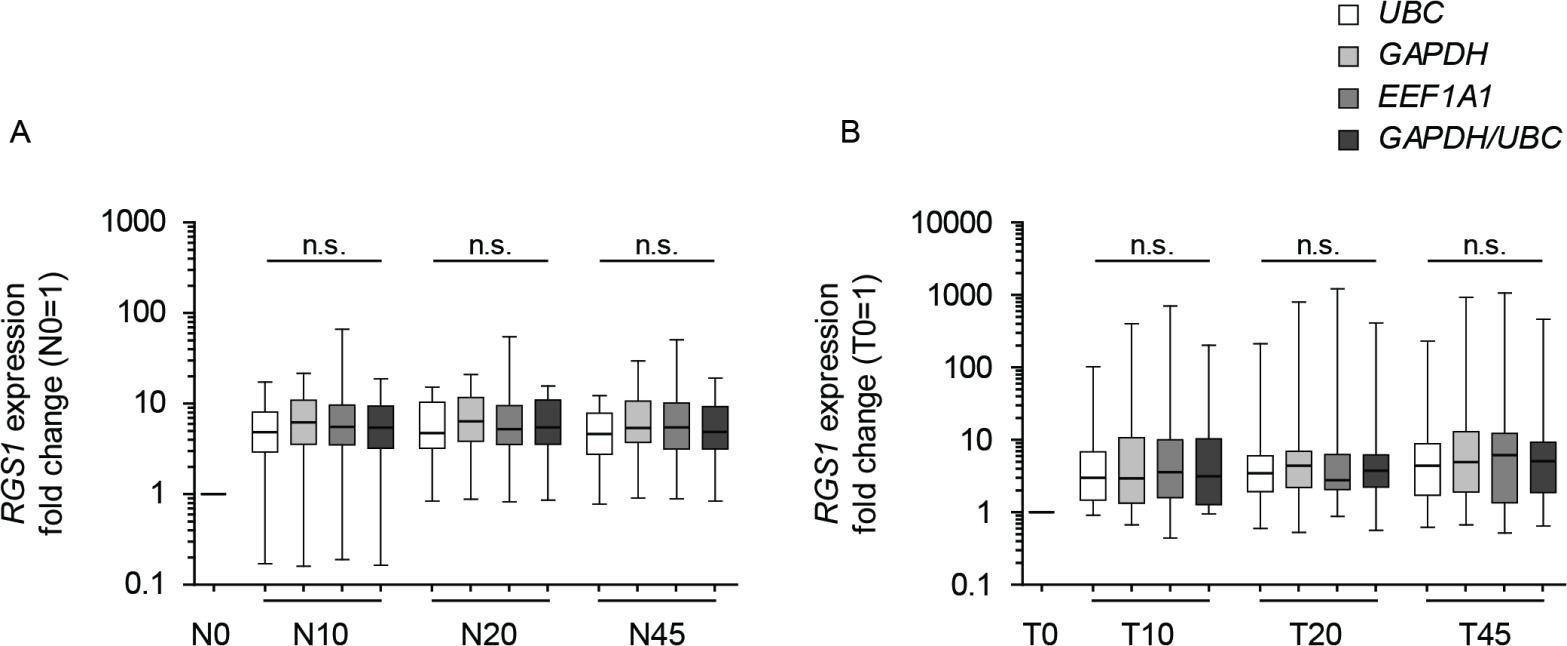
Expression levels of *RGS1* following normalization to *UBC*, *GAPDH* and *EEF1A1* as well as to the combination of *GAPDH* and *UBC*. Comparison of *RGS1* gene expression changes in normal **(A)** and tumor **(B)** ischemic tissue samples of 20 individual patients. Patients pre-surgery samples (0) were set to 1 and individual fold changes of ischemic samples were calculated based on normalization to *UBC* (white box), *GAPDH* (light grey box), *EEF1A1* (grey box) or to a combination of *GAPDH* and *UBC* (dark grey box). Results were summarized by Box-Whisker Plots. Boxes represent first and third quartile, whiskers represent minima and maxima, solid lines within boxes indicate medians. Kruskal-Wallis test and Dunn’s multiple comparison test were used for statistical analysis to compare the four normalization approaches within each time point. n.s. = not significant; N = normal tissue; T = tumor tissue; 0 = before surgery; 10, 20, 45 = 10, 20, 45 minutes after resection.

Next, all *RGS1* data were normalized to *UBC*, *GAPDH* or *EEF1A1*. *RGS1* expression level changes were compared with *RGS1* expression levels following normalization to the combination of *GAPDH* and *UBC* (**Fig 4**). Results revealed that the same *RGS1* expression levels were generated regardless of the reference gene used. Furthermore, statistical analysis revealed no significant differences in *RGS1* expression when comparing the four normalization approaches within each time point. These data support the assumption that *EEF1A1* might act as a potential reference gene of post-resectional tissue quality.

## Discussion

Biomarkers are of great interest in clinical practice not only to detect disease, but also to target the most appropriate treatment to the disease. Due to the increasing incidence of some cancers and the heterogeneity of tumors, it is important to identify biomarkers for tumor detection and classification and to aid in the development and targeting of new therapies **[1]**. The successful identification of cancer biomarkers depends on the availability of human tissue samples and the method of collection. It is well known that gene, as well as protein, expression changes in response to tissue resection and/or ischemia, therefore the time taken to collect tissue samples is of great importance **[2,3]**. To find accurate cancer biomarkers instead of ischemic artifacts, and to avoid misinterpretation of data, it is necessary to understand tissue quality. A reliable marker of tissue quality would also help to evaluate surgical samples for integration into research studies. In this study, the post-operative effects of cold ischemia on normal and CRC tissue samples were examined. This was achieved by evaluating microarray results and subsequently validating selected target gene expression by qPCR to identify a post-operative marker of tissue quality.

The results presented here demonstrate a link between the expression of *RGS1* and the post-operative effects of ischemia. *RGS1* gene expression was found to be up-regulated both in normal and colorectal tumor tissue upon resection. *RGS1* is known to be responsible for the deactivation of G-protein signaling, but little is known about the function of *RGS1* under ischemic conditions. *RGS1* has been found to be up-regulated *in vitro* in C57Bl/6 mouse ischemic retinas compared with non-ischemic retinas **[9]** and has been shown to be inducible in response to hypoxia e.g. in Burkitt`s lymphoma cell-lines **[10,11]**. This study confirms the finding that *RGS1* is up-regulated in response to ischemia or ischemic conditions. Increased *RGS1* expression could play a role in desensitization of G protein-coupled receptor (GPCR) signaling, possibly by inhibiting G-proteins [**12,13**]. Therefore, *RGS1* might be involved in protecting cells against prolonged GPCR signaling and in regulating the cellular processes that adapt to ischemic conditions. Furthermore, within this study *EEF1A1* was identified as a potential ischemia reference gene, both in normal and tumor colorectal tissue. *EEF1A1* is part of the eukaryotic elongation factor 1 (*eEF1*) complex and is responsible for GTP-dependent delivery of aminoacyl tRNAs to the ribosome **[14]**. *EEF1A1* is known to be ubiquitously expressed and has been described as a useful reference gene for real-time PCR analysis in colon and other human tissues e.g. in myocardium, in cervical tissue as well as in normal lung and lung squamous-cell carcinoma tissue **[15–19]**. This study demonstrates that *EEF1A1* is not only stably expressed in normal and tumor colorectal tissue but also following cold ischemia. This observation might be due to the fact that cells continue protein synthesis under ischemic conditions. Consequently, proteins are generated that adapt cells to ischemic stress conditions, including heat shock proteins (HSPs), and proteins involved in glucose metabolism are newly synthesized **[20]**.

In this study, microarray results of selected genes from normal ischemic colorectal tissue samples were validated by qPCR analysis. Furthermore, qPCR results confirmed the microarray expression data of *RGS1* and *EEF1A1* in tumor ischemic colorectal tissue samples whereas differential expression of *DUSP1*, *CYR61*, *SLC6A14* and *DUOX2* in tumor ischemic colorectal tissue samples could not be validated. It is possible that this is at least in part due to the methods used in this study. In comparison to qPCR, microarray analysis is known to detect weakly expressed genes with minor sensitivity, suggesting differences in expression even if the gene is not or only slightly expressed **[21,22]**. By comparing absolute qPCR expression values (Cq values), low expression levels of *SLC6A14* and *DUOX2* were observed. Furthermore, microarray results of all analyzed genes exhibited lower log-fold changes in tumor tissue samples compared with normal tissue samples. Both facts might explain the observation that qPCR results only revealed trends, not significant changes, in the expression levels of *DUSP1*, *CYR61*, S*LC6A14* and *DUOX2* in tumor ischemic colorectal tissue samples. In addition, the tumor content of samples was adjusted to ≥50% for qPCR analysis whereas such adjustment was not performed for microarray analysis. It might be possible that an increased amount of normal tissue in the microarray study hid tumor-specific effects that were not confirmable in qPCR analysis of adjusted tumor ischemic colorectal tissue samples.

Several studies have shown that pre-analytical conditions, including sample collection time, ischemic effects and the surgical collection procedure influences the molecular composition of cells. In this regard, it has been published that post-resectional storage conditions, including time and temperature, strongly affect gene expression in renal tumor tissue. The group concluded that increasing storage temperatures let to an increased number of regulated genes and this also correlated with increasing storage time **[4]**. Furthermore, Spruessel and colleagues have been shown that up to 20% of genes in healthy colorectal tissue were differentially expressed within 30 minutes of cold ischemia **[3]**. The results of the current study further underline the impact of tissue ischemia time on gene expression. More recently, it has been reported that in colorectal tissue, 10% of genes were differentially regulated 30 to 120 minutes post-resection, but clustering analysis of tumor samples from cases and time points did not reveal a set of similarly regulated genes across all cases **[23]**. Within the current study, hierarchical clustering and ANOVA analysis revealed similar changes in gene expression across both normal and tumor samples although gene expression changes were generally lower in tumor compared with normal tissue. Differences in the outcome of clustering might be due to different approaches used for clustering. More likely, differences could be due to sample collection. Yamagishi and colleagues extracted all samples including time point zero from post-surgical tissue, but within this study a colonoscopy sample served as a pre-surgical time point **[23]**.

To further evaluate these findings, a cohort of more than 20 patients should be analyzed. Future studies should also include the detection of patients’ individual gene expression levels (copy numbers) to determine cut-off values (reference ranges) in order to distinguish between fresh and ischemic tissue. Examining additional ischemic time points (e.g. 60, 90 and 120 minutes) would also be worthwhile in order to investigate possible changes over a prolonged period. Finally, within the current study, colorectal tissue was examined, however to confirm *RGS1* and *EEF1A1* as universal mRNA biomarkers a broad spectrum of tissues have to be tested to investigate the same ischemia-dependent mechanisms.

In conclusion, results from the current study revealed that post-operative ischemia significantly alters gene expression in normal and CRC tissue samples. Both microarray analysis and qPCR revealed regulator of G-protein signaling 1 (*RGS1)* as a potential marker of CRC tissue quality and eukaryotic translation elongation factor 1 alpha 1 (*EEF1A1)* as a potential reference gene of post-operative tissue quality. These findings add to our understanding of the impact of ischemic-stress on tissue sample quality, and provide an important basis for future research.

## Supporting Information

S1 Appendix Excel spreadsheet containing the numerical data and statistical analysis of Figs 1, 2A–2F, 3A–3D, 4A, 4B, S1A and S1B. (XLSX)

S1 Fig RNA integrity. The RNA integrity number (RIN) was measured using an Agilent bioanalyzer and individual RIN values are shown of all normal (A) and tumor (B) ischemic tissue samples used in qPCR analysis. Statistical analysis was performed and significant changes are indicated. Mean RIN values are displayed underlined. N = normal tissue; T = tumor tissue; 0 = before surgery; 10, 20, 45 = 10, 20, 45 minutes after resection; * p ≤ 0.05; ** p ≤ 0.01
(TIF)

S1 Table MIQE checklist for authors, reviewers and editors. All essential information (E) must be submitted with the manuscript. Desirable information (D) should be submitted if available.
(DOCX)

S2 Table qPCR validation information. (DOCX)

S3 Table qPCR target information. (DOCX)

S4 Table Clinical data of patients. Clinical data of 40 patients analyzed within the microarray study. Patients highlighted in bold were analyzed in the current qPCR study. a = Alten Eichen-Hospital Hamburg; b = Israelite Hospital Hamburg; f = female; m = male
(DOCX)
