# Supplementary figures and images for: Identification and Validation of a Potential Marker of Tissue Quality Using Gene Expression Analysis of Human Colorectal Tissue
Source: PLoS One. 2015 Jul 29;10(7):e0133987. doi: 10.1371/journal.pone.0133987 (PMC4519187; doi:10.1371/journal.pone.0133987)

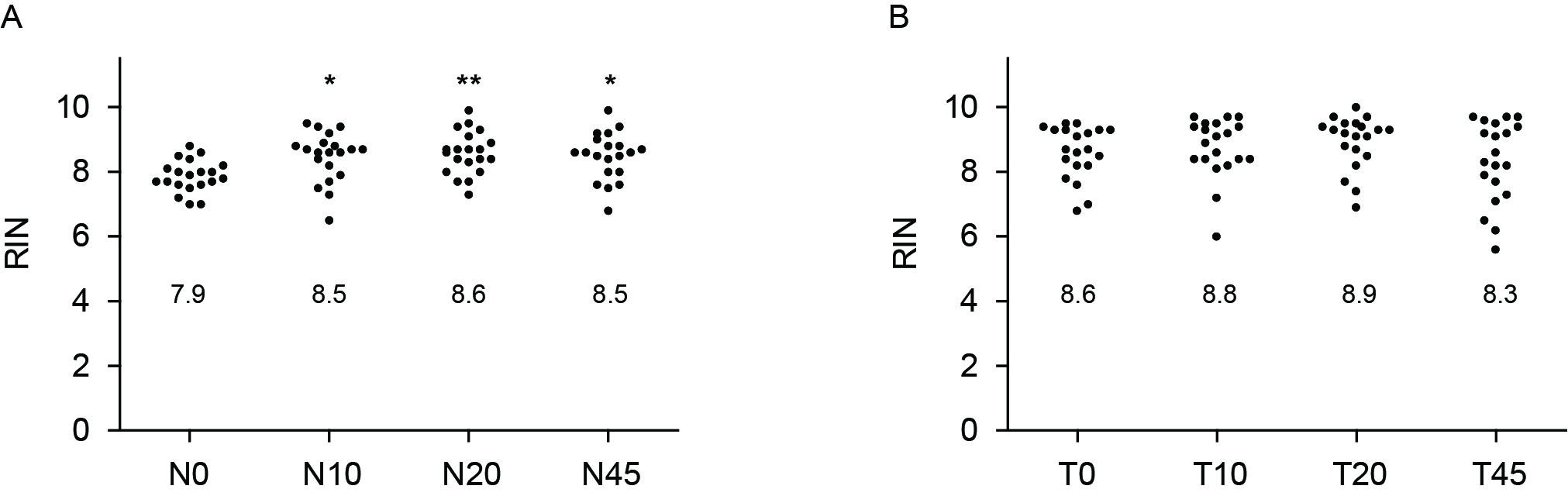

Supplement: S1 Fig — The RNA integrity number (RIN) was measured using an Agilent bioanalyzer and individual RIN values are shown of all normal (A) and tumor (B) ischemic tissue samples used in qPCR analysis. Statistical analysis was performed and significant changes are indicated. Mean RIN values are displayed underlined. N = normal tissue; T = tumor tissue; 0 = before surgery; 10, 20, 45 = 10, 20, 45 minutes after resection; * p ≤ 0.05; ** p ≤ 0.01 (TIF) [file pone.0133987.s002.tif]
