# Supplementary material for: Identification and Validation of a Potential Marker of Tissue Quality Using Gene Expression Analysis of Human Colorectal Tissue
Source: PLoS One. 2015 Jul 29;10(7):e0133987. doi: 10.1371/journal.pone.0133987 (PMC4519187; doi:10.1371/journal.pone.0133987)
Supplement: S1 Table — All essential information (E) must be submitted with the manuscript. Desirable information (D) should be submitted if available. (DOCX) [file pone.0133987.s003.docx]

S1 Table. MIQE checklist for authors, reviewers and editors.

|  |  |  |
| --- | --- | --- |
| **Item to check** | **Importance** | **Checklist** |
|  |  |  |
| **Experimental Design** |  |  |
| Definition of experimental and control groups | **E** | 20 cases for 4 ischemia time points from normal and corresponding tumor colon tissue |
| Number within each group | **E** | 8 groups, each containing 20 cases |
| Assay carried out by core lab or investigator's lab? | D |  |
| Acknowledgement of authors' contributions | D |  |
| **Sample** |  |  |
| Description | **E** | Tissue samples (colorectal) were collected from 20 patients and processed. Samples from normal tissue and tumor tissue were collected at each specified time point. |
| Volume/mass of sample processed | D | 120 - 200 mg or a size of at least 5 x 5 x 5 mm |
| Microdissection or macrodissection | **E** | Macrodissection |
| Processing procedure | **E** | Normal tissue and tumor tissue were collected at each specified time point. Each tissue sample had a weight between 120 - 200 mg or a size of at least 5 x 5 x 5 mm (=125 mm3 = 0.125 cm3). Resected tissue samples were frozen in liquid nitrogen at the indicated time points. |
| If frozen - how and how quickly? | **E** | Resected tissue samples were immediatly frozen in liquid nitrogen at the 4 ischemia time points |
| If fixed - with what, how quickly? | **E** | Not fixed |
| Sample storage conditions and duration (especially for FFPE samples) | **E** | Frozen in liquid nitrogen until RNA preparation |
| **Nucleic Acid Extraction** |  |  |
| Procedure and/or instrumentation | **E** | 1. phenol-chloroform extraction (1ml QIAzol/ sample), 2. DNase digestion, 3. RNeasy MinElute Cleanup Kit according to the supplier’s instructions |
| Name of kit and details of any modifications | **E** | QIAzol® Lysis reagent, RNase-Free DNase Set, RNeasy MinElute Cleanup Kit (Qiagen, Hilden) |
| Source of additional reagents used | D | Chloroform, Isopropanol (Sigma, Steinheim) |
| Details of DNase or RNAse treatment | **E** | in solution digestion of genomic DNA; 87.5µl RNA solution (RNA content ≤ 45 µg) + 10 µl Buffer RDD + 2.5 μl DNase I stock solution, incubation 10min room temperature (~2.7 Kunitz per 100 µl) |
| Contamination assessment (DNA or RNA) | **E** | non reverse transcriptase controls (NRTC) were performed in qPCR and no Cq values or Cq ≥ 37 were detected |
| Nucleic acid quantification | **E** | Measurement of OD260 |
| Instrument and method | **E** | NanoDrop 2000 (Thermo Scientific, USA), UV-Vis spectrophotometer |
| Purity (A260/A280) | D | between 1.8 and 2.1 |
| Yield | D |  |
| RNA integrity method/instrument | **E** | Bioanalyzer 2100 (Agilent Technologies, USA) |
| RIN/RQI or Cq of 3' and 5' transcripts | **E** | RIN ≥ 6 |
| Electrophoresis traces | D |  |
| Inhibition testing (Cq dilutions, spike or other) | **E** | 10-time dilution for standard curve |
| **Reverse Transcription** |  |  |
| Complete reaction conditions | **E** | 1.step: 2 µl 7x gDNA Wipeout Buffer (for effective elimination of genomic DNA contamination)+1 µg total RNA in 12 µl Rnase-free water (Qiagen) incubation 42°C for 2 min, 2.step: samples were mixed with 1µl Quantiscript Reverse Transcriptase+4 µl 5xQuantiscript RT Buffer+1 µl RT Primer Mix incubation 42°C for 15 min followed by 95°C for 3 min on iCycler Thermal Cycler (Biorad, Munich) |
| Amount of RNA and reaction volume | **E** | Amount: 1 µg RNA, Volume: 20 µl |
| Priming oligonucleotide (if using GSP) and concentration | **E** | 1 µl RT Primer Mix (Qiagen, Hilden): optimized blend of oligo-dT and random primers dissolved in water (Qiagen, Hilden) |
| Reverse transcriptase and concentration | **E** | 1 µl of Quantiscript® Reverse Transcriptase: A mixture of the QIAGEN® products Omniscript® Reverse Transcriptase and Sensiscript® Reverse Transcriptase. Also contains RNase inhibitor (Qiagen, Hilden) |
| Temperature and time | **E** | specified in "Complete reaction conditions" |
| Manufacturer of reagents and catalogue numbers | D | QuantiTec Reverse Transcription Kit (Cat.No: 205311, Qiagen, Hilden) |
| Cqs with and without RT | D* | Cq without RT ≥ Cq with RT + 10 |
| Storage conditions of cDNA | D | -20°C |
| **qPCR Target Information** |  |  |
| If multiplex, efficiency and LOD of each assay. | **E** | Not applicable |
| Sequence accession number | **E** | S3 Table |
| Location of amplicon | D | S3 Table |
| Amplicon length | **E** | S3 Table |
| *In silico* specificity screen (BLAST, etc) | **E** | Yes, but not applicable but Biorad key requirements for primer design were published in "PrimePCR_Assay_Validation_Tech_Note_6262" |
| Pseudogenes, retropseudogenes or other homologs? | D | not applicable but Biorad key requirements for primer design were published in "PrimePCR_Assay_Validation_Tech_Note_6262" |
| Sequence alignment | D | Yes, but not applicable but Biorad key requirements for primer design were published in "PrimePCR_Assay_Validation_Tech_Note_6262" |
| Secondary structure analysis of amplicon | D | Yes, but not applicable but Biorad key requirements for primer design were published in "PrimePCR_Assay_Validation_Tech_Note_6262" |
| Location of each primer by exon or intron (if applicable) | **E** | S3 Table |
| What splice variants are targeted? | **E** | Not applicable but Biorad key requirements for primer design were published in "PrimePCR_Assay_Validation_Tech_Note_6262" |
| **qPCR Oligonucleotides** |  |  |
| Primer sequences | **E** | Not applicable, but Biorad Unique Assay ID is listed in S3 Table |
| RTPrimerDB Identification Number | D |  |
| Probe sequences | D** | S3 Table, Biorad provided Amplicon Context Sequence |
| Location and identity of any modifications | **E** | No modifications |
| Manufacturer of oligonucleotides | D | Biorad, Hilden |
| Purification method | D | Desalted |
| **qPCR Protocol** |  |  |
| Complete reaction conditions | **E** | Reaction: 2 µl cDNA (1/10 dilution of original cDNA) + 7 µl RNase free water + 10 µl 2x SsoAdvanced universal SYBR® Green supermix (Biorad, Hilden) + 1 µl 20x PrimePCR™ SYBR® Green Assay (Primer, Biorad, Hilden). The thermal cycling protocol included a single polymerase activation step at 95 °C for 2 min followed by 40 amplification cycles as well as melt curve analysis. Each amplification cycle implied a denaturation step at 95 °C for 10 sec and a primer annealing/ elongation step at 60 °C for 30 sec. Melt curve analysis included a 30 sec hold at 65 °C followed by a gradual increase to 95 °C with a temperature increment of 0.5°C/ 5 sec. Samples as well as the positive control were measured in triplicates whereas non-template controls (NTC) and non reverse transcriptase controls (NRTC) were measured in duplicates. |
| Reaction volume and amount of cDNA/DNA | **E** | Amplification of cDNA generated from 10 ng of RNA (2 µl of a 1/10 dilution of original cDNA) in a reaction volume of 20 µl |
| Primer, (probe), Mg++ and dNTP concentrations | **E** | PrimePCR™ SYBR® Green Assay |
| Polymerase identity and concentration | **E** | 2x SsoAdvanced universal SYBR® Green supermix (Cat.No: 172-5271, Biorad, Hilden): contains antibody-mediated hot-start Sso7d fusion polymerase, concentration not specified |
| Buffer/kit identity and manufacturer | **E** | 2x SsoAdvanced universal SYBR® Green supermix (Cat.No: 172-5271, Biorad, Hilden): contains antibody-mediated hot-start Sso7d fusion polymerase, dNTPs, MgCl2, SYBR® Green I dye, enhancers, stabilizers, and a blend of passive refence dyes (including ROX and fluorescein), no concentrations specified |
| Exact chemical constitution of the buffer | D | No informations specified |
| Additives (SYBR Green I, DMSO, etc.) | **E** | 2x SsoAdvanced universal SYBR® Green supermix (Cat.No: 172-5271, Biorad, Hilden): SYBR® Green I dye, enhancers, stabilizers, and a blend of passive refence dyes (including ROX and fluorescein), not specified |
| Manufacturer of plates/tubes and catalog number | D | Hard-Shell® Low-Profile Thin-Wall 96-Well Skirted PCR Plates (Cat.No: HSP-9601, Biorad, Hilden) |
| Complete thermocycling parameters | **E** | 95 °C for 2 min; 40x 95 °C for 10 sec + 60 °C for 30 sec; 65 °C 30 sec followed by a gradual increase to 95 °C (increment of 0.5°C/ 5 sec) |
| Reaction setup (manual/robotic) | D | Manual pipetting |
| Manufacturer of qPCR instrument | **E** | C1000 Touch™ Thermal Cycler + Bio-Rad CFX Manager™ Software Version 3.0 (Biorad, Hilden) |
| **qPCR Validation** |  |  |
| Evidence of optimisation (from gradients) | D |  |
| Specificity (gel, sequence, melt, or digest) | **E** | Melt temperature ± 1°C based on published data of Biorads PrimePCR™ SYBR® Green Assays (Biorad, Hilden), no melt curve of non-template controls (NTC) and non reverse transcriptase controls (NRTC) |
| For SYBR Green I, Cq of the NTC | **E** | No Cq value of NTC |
| Standard curves with slope and y-intercept | **E** | S2 Table |
| PCR efficiency calculated from slope | **E** | S2 Table |
| Confidence interval for PCR efficiency or standard error | D |  |
| r2 of standard curve | **E** | S2 Table |
| Linear dynamic range | **E** | S2 Table |
| Cq variation at lower limit | **E** | S2 Table |
| Confidence intervals throughout range | D |  |
| Evidence for limit of detection | **E** | S2 Table |
| If multiplex, efficiency and LOD of each assay. | **E** | Not applicable |
| **Data Analysis** |  |  |
| qPCR analysis program (source, version) | **E** | Bio-Rad CFX Manager™ Software Version 3.0 (Biorad, Hilden) |
| Cq method determination | **E** | Auto-calculated generation of threshold by CFX Manager™ Software Version 3.0 (Biorad, Hilden) |
| Outlier identification and disposition | **E** | Triplicates: exclusion of max. one value |
| Results of NTCs | **E** | Non-template controls = Cq ≥ 37 |
| Justification of number and choice of reference genes | **E** | 2 reference genes known to be constantly expressed in colon tissue |
| Description of normalisation method | **E** | ∆∆Cq method; target gene expression levels were normalized to the mean of reference gene expression levels |
| Number and concordance of biological replicates | D | No biological replicates |
| Number and stage (RT or qPCR) of technical replicates | **E** | Samples as well as the positive control (qPCR) were measured in triplicates whereas non-template controls (NTC) and non reverse transcriptase controls (NRTC) were measured in duplicates in the same run. |
| Repeatability (intra-assay variation) | E | Standard deviation of triplicates = SD ≤ 0.5 |
| Reproducibility (inter-assay variation, %CV) | D | CV ≤ 5.23 %, reproducibility ≥ 94.77 % |
| Power analysis | D |  |
| Statistical methods for result significance | **E** | Kruskal-Wallis Test and Dunn`s Multiple Comparison Test |
| Software (source, version) | E | GraphPad Prism 5 (GraphPad Software, Inc.; La Jolla, USA) |
| Cq or raw data submission using RDML | **D** |  |
| **qPCR Oligonucleotides** |  |  |
| Primer sequences | **E** | Not applicable, but Biorad Unique Assay ID is listed in S3 Table |
| RTPrimerDB Identification Number | D |  |

All essential information (E) must be submitted with the manuscript. Desirable information (D) should be submitted if available.
