# Supplementary material for: Identification and Validation of a Potential Marker of Tissue Quality Using Gene Expression Analysis of Human Colorectal Tissue
Source: PLoS One. 2015 Jul 29;10(7):e0133987. doi: 10.1371/journal.pone.0133987 (PMC4519187; doi:10.1371/journal.pone.0133987)
Supplement: S2 Table — (DOCX) [file pone.0133987.s004.docx]

S2 Table. qPCR validation information.

| **Biorad Unique Assay ID** | **gene symbol** | **positive control** | **linear dynamic range (cDNA generated from RNA)** | **PCR product length, bp** | **Efficiency** | **slope** | **R^2^** | **y intercept** | **Evidence for limit of detection (cDNA generated from RNA)** | **Cq variation at lower limit (Cq difference between triplicates)** |
| --- | --- | --- | --- | --- | --- | --- | --- | --- | --- | --- |
| qHsaCED0036260 | *GAPDH* | MDA-MB-231 | 100-0.001 | 61 | 104.4 | -3.221 | 0.999 | 23.75 | ≤ 0.001 | ≥ 1 |
| qHsaCED0038674 | *GAPDH* | HeLa | 10-0.0001 | 117 | 97 | -3.397 | 0.999 | 20.144 | ≤ 0.0001 | ≥ 1 |
| qHsaCED0023867 | *UBC* | MDA-MB-231 | 10-0.001 | 119 | 99.9 | -3.324 | 0.997 | 24.407 | ≤ 0.001 | ≥ 1 |
| qHsaCED0001963 | *CYR61* | MDA-MB-231 | 100-0.01 | 83 | 104.4 | -3.222 | 0.997 | 24.992 | ≤ 0.01 | ≥ 1 |
| qHsaCID0015361 | *DUOX2* | Human DUOX2 PrimePCR™ Template | 20000000-20 copies | 93 | 97.5 | -3.384 | 1.000 | 40.786 | ≤ 20 copies | ≥ 1 |
| qHsaCID0023130 | *RGS1* | Human RGS1 PrimePCR™ Template | 20000000-20 copies | 123 | 102.1 | -3.273 | 1.000 | 38.410 | ≤ 20 copies | ≥ 1 |
| qHsaCED0020436 | *EEF1A1* | Lovo | 10-0.0032 | 99 | 95.9 | -3.425 | 0.997 | 26.236 | ≤ 0.0032 | ≥ 1 |
| qHsaCID0008599 | *SLC6A14* | MCF7 | 20-0.032 | 74 | 95.7 | -3.429 | 0.996 | 28.582 | ≤ 0.032 | ≥ 1 |
| qHsaCED0004747 | *DUSP1* | HeLa | 20-0.000256 | 60 | 103.1 | -3.250 | 0.998 | 24.909 | ≤ 0.000256 | ≥ 1 |
