# Supplementary material for: Identification and Validation of a Potential Marker of Tissue Quality Using Gene Expression Analysis of Human Colorectal Tissue
Source: PLoS One. 2015 Jul 29;10(7):e0133987. doi: 10.1371/journal.pone.0133987 (PMC4519187; doi:10.1371/journal.pone.0133987)
Supplement: S3 Table — (DOCX) [file pone.0133987.s005.docx]

S3 Table. qPCR target information.

| **Biorad Unique Assay ID** | **gene symbol** | **Sequence accession number** | **UniGene ID** | **Location of amplicon** | **Amplicon length, bp** | **Amplicon Context Sequence** | **Location of each primer by exon or intron** |
| --- | --- | --- | --- | --- | --- | --- | --- |
| qHsaCED0036260 | *GAPDH* | NC_000012.11, NG_007073.2, NT_009759.16 | Hs.544577 | 12:6646937-6647027 | 61 | CCAACGTGTCAGTGGTGGACCTGACCTGCCGTCTAGAAAAACCTGCCAAATATGATGACATCAAGAAGGTGGTGAAGCAGGCGTCGGAGGG | exonic |
| qHsaCED0038674 | *GAPDH* | NC_000012.11, NG_007073.2, NT_009759.16 | Hs.544577 | 12:6647267-6647413 | 117 | GTATGACAACGAATTTGGCTACAGCAACAGGGTGGTGGACCTCATGGCCCACATGGCCTCCAAGGAGTAAGACCCCTGGACCACCAGCCCCAGCAAGAGCACAAGAGGAAGAGAGAGACCCTCACTGCTGGGGAGTCCCTGCCACAC | exonic |
| qHsaCED0023867 | *UBC* | *NC_000012.11, NG_027722.1, NT_009755.19* | Hs.520348 | 12:125397941-125398089 | 119 | AGCTGCTTTCCGGCAAAGATCAACCTCTGCTGGTCAGGAGGAATGCCTTCCTTGTCCTGGATCTTTGCTTTGACGTTCTCGATGGTGTCACTGGGCTCGACCTCAAGGGTGATGGTCTTGCCAGTGAGTGTCTTCACGAAGATTTGCAT | exonic |
| qHsaCED0001963 | *CYR61* | *NC_000001.10, NT_032977.9* | Hs.8867 | 1:86047790-86047902 | 83 | GTTACCGGGCAGTGCTGCGAGGAGTGGGTCTGTGACGAGGATAGTATCAAGGACCCCATGGAGGACCAGGACGGCCTCCTTGGCAAGGAGCTGGGATTCGATGCCTCCGAGGT | exonic |
| qHsaCID0015361 | *DUOX2* | *NC_000015.9, NG_009447.1, NG_016992.1, NT_010194.17* | Hs.71377 | 15:45394134-45396226 | 93 | ACCTCGGCCAGCTGGGCCTTGGACAGGCAGTTGTTGGAGATCTCGATGAAGGATCGCATCATGGTGAAGAATTCGTCCTTGGAGAGGAAGCCATTCTCATCCAGGTCATACATGGTAAACATT | intron-spanning |
| qHsaCID0023130 | *RGS1* | *NC_000001.10, NT_004487.19* | Hs.75256 | 1:192547408-192548311 | 123 | GAGTTCTGGCTGGCTTGTGAAGACTATAAGAAAACAGAGTCTGATCTTTTGCCCTGTAAAGCAGAAGAGATATATAAAGCATTTGTGCATTCAGATGCTGCTAAACAAATCAATATTGACTTCCGCACTCGAGAATCTACAGCCAAGAAGATT | intron-spanning |
| qHsaCED0020436 | *EEF1A1* | *NC_000006.11, NT_007299.13* | Hs.586423 | 6:74226599-74226727 | 99 | CTAATTATCAAAGCATAAAAGGTATTAGACTCTGCAGGAGAAAAGCAATGTAGATTAGTCTAATTTTATAGCTACTTCAAATTGCCATCTTTTTCTATTAGAACCTTGTTCCTATTCTGAATAGCACTC | exonic |
| qHsaCID0008599 | *SLC6A14* | *NC_000023.10, NG_021305.1, NT_028405.12* | Hs.522109 | X:115567869-115569004 | 74 | GAGTGAACCATGGACAAGTTGAAATGCCCGAGTTTCTTCAAGTGCAGGGAGAAGGAGAAAGTGTCGGCTTCATCAGAGAATTTCCATGTTGGTGAAAATGATGA | intron-spanning |
| qHsaCED0004747 | *DUSP1* | *NC_000005.9, NT_023133.13* | Hs.171695 | 5:172196629-172196718 | 60 | TGTCCTCCACAGGGATGCTCTTGTACTGGTAGTGACCCTCAAAATGGTTGGGACAATTGGCTGAGACGTTGATCAAGGCAGTGATGCCCA | exonic |
