# Supplementary material for: Identification and Validation of a Potential Marker of Tissue Quality Using Gene Expression Analysis of Human Colorectal Tissue
Source: PLoS One. 2015 Jul 29;10(7):e0133987. doi: 10.1371/journal.pone.0133987 (PMC4519187; doi:10.1371/journal.pone.0133987)
Supplement: S4 Table — Clinical data of 40 patients analyzed within the microarray study. Patients highlighted in bold were analyzed in the current qPCR study. a = Alten Eichen-Hospital Hamburg; b = Israelite Hospital Hamburg; f = female; m = male (DOCX) [file pone.0133987.s006.docx]

S4 Table. Clinical data of patients.

| **Case no.** | **Age [yrs]** | **Gender** | **Hospital** | **Description** | **Tumor size [cm]** | **Histological type** | **Grading** | **Stage** |
| --- | --- | --- | --- | --- | --- | --- | --- | --- |
| NC2 | 60 | m | a | Malignant neoplasm of rectum | 5 | Poorly differentiated adenocarcinoma | G3 | IV |
| NC3 | 68 | f | a | Malignant neoplasm of rectum | 2.5 | Poorly differentiated adenocarcinoma | G3 | IIIA |
| **NC4** | 54 | f | a | Malignant neoplasm of sigmoid colon | 1.8 | Moderately differentiated adenocarcinoma | G2 | I |
| **NC5** | 71 | m | a | Malignant neoplasm of rectum | 7 | Moderately differentiated carcinoma | G2 | IVA |
| NC7 | 73 | m | a | Malignant neoplasm of rectum | 6.5 | Moderately to poorly differentiated adenocarcinoma | G3 | IIA |
| NC8 | 80 | f | a | Malignant neoplasm of rectum | 4 | Adenoma with high-grade epith. dysplasia | n/a | n/a |
| NC9 | 88 | f | a | Malignant neoplasm of rectum | 3 | Poorly differentiated adenocarcinoma | G3 | IVA |
| NC10 | 44 | f | a | Malignant neoplasm of rectum | 4 | Moderately to poorly differentiated adenocarcinoma | G3 | IIA |
| **NC11** | 62 | m | a | Malignant neoplasm of sigmoid colon | 7 | Poorly differentiated adenocarcinoma | G3 | IIA |
| **NC12** | 66 | f | a | Malignant neoplasm of rectum | 4.5 | Poorly differentiated adenocarcinoma | G3 | IVA |
| NC13 | 56 | m | a | Malignant neoplasm of rectum | 5 | Moderately differentiated adenocarcinoma | G2 | IVA |
| NC15 | 89 | f | a | Malignant neoplasm of rectum | 4.5 | Poorly differentiated adenocarcinoma | G3 | IVA |
| **NC16** | 69 | f | a | Carcinoma in situ of rectum | 7 | Moderately differentiated adenocarcinoma | G2 | 0 |
| NC17 | 66 | m | a | Malignant neoplasm of rectum | 3.5 | Moderately differentiated mucinous adenocarcinoma | G2 | I |
| NC18 | 84 | f | a | Malignant neoplasm of rectum | 5 | Moderately differentiated adenocarcinoma | G2 | IVA |
| NC20 | 73 | m | a | Malignant neoplasm of rectum | 4.5 | Moderately differentiated adenocarcinoma | G2 | I |
| **NC21** | 69 | m | b | Malignant neoplasm of sigmoid colon | 4,5 | Moderately differentiated adenocarcinoma | G2 | IIA |
| NC22 | 47 | m | b | Malignant neoplasm of rectum | 7.5 | Moderately differentiated adenocarcinoma | G2 | IIC |
| NC23 | 48 | m | b | Malignant neoplasm of sigmoid colon | 3.5 | Moderately differentiated adenocarcinoma | G2 | IVB |
| **NC25** | 45 | m | b | Malignant neoplasm of rectum | 6 | Moderately differentiated adenocarcinoma | G2 | IIIB |
| **NC26** | 71 | f | b | Malignant neoplasm of sigmoid colon | 9 | Poorly differentiated adenocarcinoma | G3 | IVB |
| **NC27** | 64 | m | b | Malignant neoplasm of sigmoid colon | 4.5 | Moderately differentiated adenocarcinoma | G2 | n/a |
| NC28 | 56 | m | b | Malignant neoplasm of rectum | 5 | Moderately differentiated adenocarcinoma | G2 | IIIB |
| NC29 | 67 | f | b | Malignant neoplasm of sigmoid colon | 3 | Moderately differentiated adenocarcinoma | G2 | IIIB |
| **NC30** | 71 | f | b | Malignant neoplasm of sigmoid colon | 7 | Moderately differentiated adenocarcinoma | G2 | I |
| NC32 | 56 | m | b | Malignant neoplasm of sigmoid colon | 5.5 | Poorly differentiated mucinous adenocarcinoma | G3 | IIIC |
| NC33 | 59 | m | b | Malignant neoplasm of rectum | 3.5 | Moderately differentiated adenocarcinoma | G2 | IVA |
| NC34 | 67 | f | b | Malignant neoplasm of sigmoid colon | 3 | Moderately differentiated adenocarcinoma | G2 | IIIB |
| **NC35** | 74 | f | b | Malignant neoplasm of descending colon | 3 | Moderately differentiated, partially mucinous adenocarcinoma | G2 | IIIA |
| **NC36** | 61 | f | b | Malignant neoplasm of sigmoid colon | 4.5 | Moderately differentiated adenocarcinoma | G2 | I |
| **NC37** | 65 | f | b | Malignant neoplasm of rectum | 5 | Poorly differentiated adenocarcinoma | G3 | I |
| NC38 | 76 | f | b | Malignant neoplasm of rectum | 4.5 | Poorly differentiated mucinous adenocarcinoma | G3 | IIA |
| **NC39** | 70 | m | b | Malignant neoplasm of rectum | 6 | Moderately differentiated mucinous adenocarcinoma | G2 | IIA |
| **NC40** | 66 | f | b | Malignant neoplasm of rectum | 6 | Moderately differentiated adenocarcinoma | G2 | I |
| **NC41** | 88 | m | b | Malignant neoplasm of rectum | 3.5 | Moderately differentiated adenocarcinoma | G2 | IVA |
| **NC42** | 76 | m | b | Malignant neoplasm of sigmoid colon | 3 | Poorly differentiated adenocarcinoma | G3 | III B |
| **NC43** | 72 | f | b | Malignant neoplasm of sigmoid colon | 3.5 | Moderately differentiated adenocarcinoma | G2 | IIA |
| NC46 | 53 | f | b | Malignant neoplasm of sigmoid colon | 4 | Moderately differentiated adenocarcinoma | G2 | IIA |
| **NC47** | 83 | f | b | Malignant neoplasm of sigmoid colon | 4 | Moderately differentiated adenocarcinoma | G2 | I |
| **NC48** | 74 | m | b | Malignant neoplasm of sigmoid colon | 4.5 | poorly differentiated mucinous adenocarcinoma | G3 | IIA |

Clinical data of 40 patients analyzed within the microarray study. Patients highlighted in bold were analyzed in the current qPCR study. a = Alten Eichen-Hospital Hamburg; b = Israelite Hospital Hamburg; f = female; m = male
